# Superelastic and Washable Micro/Nanofibrous Sponges Based on Biomimetic Helical Fibers for Efficient Thermal Insulation

**DOI:** 10.1007/s40820-025-01882-2

**Published:** 2025-08-25

**Authors:** Fengjin Yang, Zhifei Wang, Wei Zhang, Sai Wang, Yi-Tao Liu, Fei Wang, Roman A. Surmenev, Jianyong Yu, Shichao Zhang, Bin Ding

**Affiliations:** 1 Innovation Center for Textile Science and Technology, College of Textiles, Donghua University, Shanghai, 200051 People’s Republic of China; 2 School of Materials Science and Engineering, Shanghai University of Engineering Science, Shanghai, 201620 People’s Republic of China; 3 Physical Materials Science and Composite Materials Center, Research School of Chemistry & Applied Biomedical Sciences, National Research Tomsk Polytechnic University, Tomsk, Russia 634050

**Keywords:** Electrospinning, Micro/nanofibrous sponge, Hierarchical structure, Superelasticity, Thermal insulation

## Abstract

Extreme cold weather seriously harms human thermoregulatory system, necessitating high-performance insulating garments to maintain body temperature. However, as the core insulating layer, advanced fibrous materials always struggle to balance mechanical properties and thermal insulation, resulting in their inability to meet the demands for both washing resistance and personal protection. Herein, inspired by the natural spring-like structures of cucumber tendrils, a superelastic and washable micro/nanofibrous sponge (MNFS) based on biomimetic helical fibers is directly prepared utilizing multiple-jet electrospinning technology for high-performance thermal insulation. By regulating the conductivity of polyvinylidene fluoride solution, multiple-jet ejection and multiple-stage whipping of jets are achieved, and further control of phase separation rates enables the rapid solidification of jets to form spring-like helical fibers, which are directly entangled to assemble MNFS. The resulting MNFS exhibits superelasticity that can withstand large tensile strain (200%), 1000 cyclic tensile or compression deformations, and retain good resilience even in liquid nitrogen (− 196 °C). Furthermore, the MNFS shows efficient thermal insulation with low thermal conductivity (24.85 mW m^−1^ K^−1^), close to the value of dry air, and remains structural stability even after cyclic washing. This work offers new possibilities for advanced fibrous sponges in transportation, environmental, and energy applications.

**Supplementary Information:**

The online version contains supplementary material available at 10.1007/s40820-025-01882-2.

## Highlights

- A superelastic and washable sponge based on biomimetic spring-like helical micro/nanofibers is directly fabricated by multiple-jet electrospinning technology.
- The resulting sponge exhibits both lightweight (low density of 7.1 mg cm^–3^) and robust mechanical property (large tensile strain up to 200%).
- The sponge also shows efficient thermal insulation performance with low thermal conductivity (24.85 mW m^–1^ K^–1^), and remains structural stability even after cyclic washing, making it a promising candidate for personal protection in cold environments.

**Supplementary Information:**

The online version contains supplementary material available at 10.1007/s40820-025-01882-2.

## Graphical abstract

**Figure:**
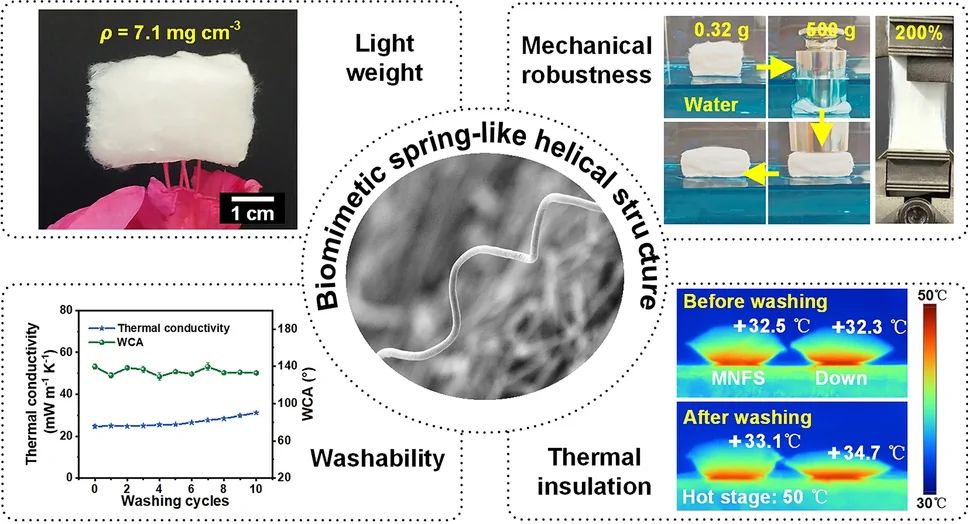

## Introduction

Humans are warm-blooded animals that rely on their internal thermoregulatory system to maintain body temperature, while extreme cold weather can lead to hypothermia or frostbite [1]. Therefore, maintaining body temperature in extreme environments is essential to ensure human health [2–4]. Typically, measures to keep warm in cold conditions include indoor heating by air conditioning or wearing cold-protective clothing [5, 6]. Although air conditioning is a common method, it consumes excessive amounts of energy, putting significant pressure on energy supply and environmental conservation [7, 8]. Additionally, the use of complex and heavy indoor heating equipment cannot satisfy the requirements for keeping human body warm in constantly changing outdoor surroundings. On the other hand, cold-protective clothing, acting as a second skin, has been the primary mediator for thermoregulation [9, 10]. The core thermal insulation layer of these garments is always made of fibrous materials, including natural and synthetic fibers. Natural fibers, such as wool, down, and cotton, have become well-known thermal insulation materials due to their skin-friendly properties [11]. However, these materials are easy to absorb moisture, which leads to a significant decrease in structure stability and thermal insulation performance after washing. In contrast, synthetic fibers have advantages such as cost-effectiveness, low thermal conductivity, and moisture resistance, making them a potential replacement for natural fibers. Unfortunately, synthetic fibers still face the defects such as large diameter (generally > 10 μm) and restricted porosity, leading to limited improvement in thermal insulation performance. Moreover, the conventional method for achieving efficient thermal insulation is to make fibrous materials thicker and heavier, which can be uncomfortable for the wearer. Therefore, developing lightweight and efficient fibrous thermal insulation materials is crucial.

Electrospun fibers possess desirable structural characteristics, including small diameter, low density, and small pore size, which make them potential for efficient thermal insulation [12–14]. However, the inherent lamellar deposition feature of electrospun fibers often results in densely packed membranes with limited porosity (< 75%) and thickness (< 50 μm), rather than three-dimensional (3D) materials [15, 16]. To overcome this problem, researchers have recently used freeze-drying technology to reconstruct the electrospun fibers into 3D networks, resulting in the formation of fibrous sponges with low density and high porosity. However, the skeleton structure of these sponges is formed by discontinuous short fibers and lacks effective entanglement between fibers, resulting in weak mechanical properties and unstable pore structure. Furthermore, the complex process and long production cycle hinder the large-scale production and application of sponges [17–19]. Recently, some fibrous sponges have been directly prepared using electrospinning, with their mechanical properties enhanced by developing highly-buckled fiber architectures or bonding points between fibers [20–22]. However, these fibers always lack elastic recovery, causing irreversible structural collapse of the sponge during washing cycles. In addition, the single-mode insulation mechanism of the fluffy sponge that relies on suppressing convective heat transfer limits further improvement in thermal insulation performance. Consequently, the construction of superelastic and washable fibrous sponges for efficient thermal insulation remains a significant challenge.

In nature, many climbing plants exhibit spontaneous coiling behavior in their tendrils (e.g., cucumber, luffa), forming spring-like helical structures [23]. The superior performance of these biological springs arises from their hierarchical helical organization, which facilitates efficient energy dissipation, strain buffering, and multi-scale pore regulation. Here, by mimicking the structural principles of cucumber tendrils, we present a scalable strategy to directly prepare superelastic and washable sponges based on biomimetic spring-like helical micro/nanofibers using multiple-jet electrospinning technology for thermal insulation. Our design manipulates the conductivity of solution and the concentration of highly volatile acetone in the mixed solvent system to achieve multiple-jet injection, multiple-stage jet whipping, rapid phase separation, and solidification of whipping jets. This results in the formation of biomimetic spring-like helical fibers with porous structures, which are directly entangled with each other to assemble a micro/nanofibrous sponge (MNFS). The obtained MNFS exhibits high stretchability with large tensile recovery strain of 200%, remarkable compressibility with low plastic deformation following 1000 cycles, and good resistance to liquid nitrogen (− 196 °C). Furthermore, the MNFS also demonstrates efficient thermal retention capacity with low thermal conductivity (24.85 mW m^−1^ K^−1^), close to the value of dry air and remains structural stability after cyclic washing. All of these properties make MNFS a promising candidate for retaining warmth in various cold conditions.

## Experimental Section

### Materials

Polyvinylidene fluoride (PVDF) powder (Mw = 570,000) was provided by Solvay Co., Ltd, USA. N, N-dimethylacetamide (DMAc) and acetone (AC) were supplied by Sinopharm Chemical Reagent Co., Ltd., China. Fluorinated polyurethane (FPU) was obtained from Shanghai Taifu Chemical Co., Ltd., China. LiCl was bought from Shanghai Aladdin Chemistry Co., Ltd.

### Preparation of Micro/Nanofibrous Sponges Based on PVDF Helical Fiber

A homogeneous spinning solution was obtained by adding 18 wt% PVDF powder to the mixed solvents of DMAc and AC (the AC concentration was 20 wt%) at 50 °C for 8 h, which contained various concentration of LiCl (0 wt%, 0.002 wt%, 0.004 wt%, and 0.006 wt%). FPU was added to the spinning solution at a concentration of 5 wt%. The LiCl concentration in the solution was then maintained constant at 0.004 wt%, while the AC concentration in the DMAc/AC mixed solvent was 20 wt%, 40 wt%, and 60 wt%, respectively. An electrospinning machine (DXES, SOF Nanotechnology Co., Ltd., China) was utilized to prepare all fibrous materials. The optimal fiber sponge was prepared from an 18 wt% solution containing 0.004 wt% LiCl and 40 wt% AC. The spinning chamber temperature and humidity were 22 ± 3 °C and 90% ± 3%, respectively. The solution was ejected at the feed rate of 5 mL h^−1^ and voltage of 30 kV. Fibrous materials were collected on a grounded metal roller (rotation speed: 10 r min^−1^), with a nozzle-to-collector distance of 23 cm.

### Characterization

The spinning solutions properties (including conductivity, viscosity, and surface tension) were characterized using a conductivity meter (FE30, Mettler-Toledo Instruments Co., Ltd., Switzerland), a rotational viscometer (LVDV-1T, Fangrui Instrument Co., Ltd., China), and a surface tension meter (QBZY, Fangrui Instrument Co., Ltd., China), respectively. The morphologies and microstructures of the prepared materials were characterized using a scanning electron microscope (SEM, SU5000, Hitachi Co., Ltd., Japan). The porous structure of fibrous materials was evaluated by a physisorption analyzer (ASAP 2460, Micromeritics Inc., USA). The specific surface area and pore size distribution were measured by the Brunauer–Emmett–Teller (BET) model and density functional theory (DFT), respectively. The porosity of fibrous materials has been analyzed by means of the formula below:

$$\mathrm{Porosity}=\left(1-\frac{\rho }{{\rho }_{0}}\right)\times 100{\%}$$

where *ρ* is the volume density of fibrous materials and *ρ*_0_ is the density of polymer. The tensile testing machine (ST390P, Suzhou Rising Intelligent Technology Co., Ltd., China) and dynamic mechanical analyzer (DMA, Q850, TA Instruments, USA) were used to study the mechanical characteristics of fibrous materials. The thermal conductivities of fibrous materials were characterized at 25 ± 2 °C and 60% ± 3% relative humidity (RH) by thermal constants analyzer (TPS2500, Hot Disk Inc., Sweden). A thermal camera (Tis75, Fluke Inc., USA) was used to take the infrared images. The temperature of fibrous assemblies in air and underwater was characterized using a multi-channel temperature recorder (MT500P, Shenzhen Shenhua Technology Co., Ltd., China). The washability of MNFS was tested by using a beaker with a magnetic stirring rotor to simulate gentle machine washing. One washing cycle consisted of: (1) Washing process: 20 min at room temperature with stirring at 800 r min^−1^ and no detergent; (2) Dry process: 1 h at 60 °C in an oven until completely dry. The WCAs were characterized by goniometer instrument (SL200B, Kino Co., Ltd., USA). The UV-resistance testing was conducted by using an Ultraviolet Transmittance Analyzer (UV-2000F, USA) over 250–400 nm according to the GB/T 18830-2009 standard.

## Results and Discussion

### Design Strategy and Preparation of MNFS

**Fig. 1:**
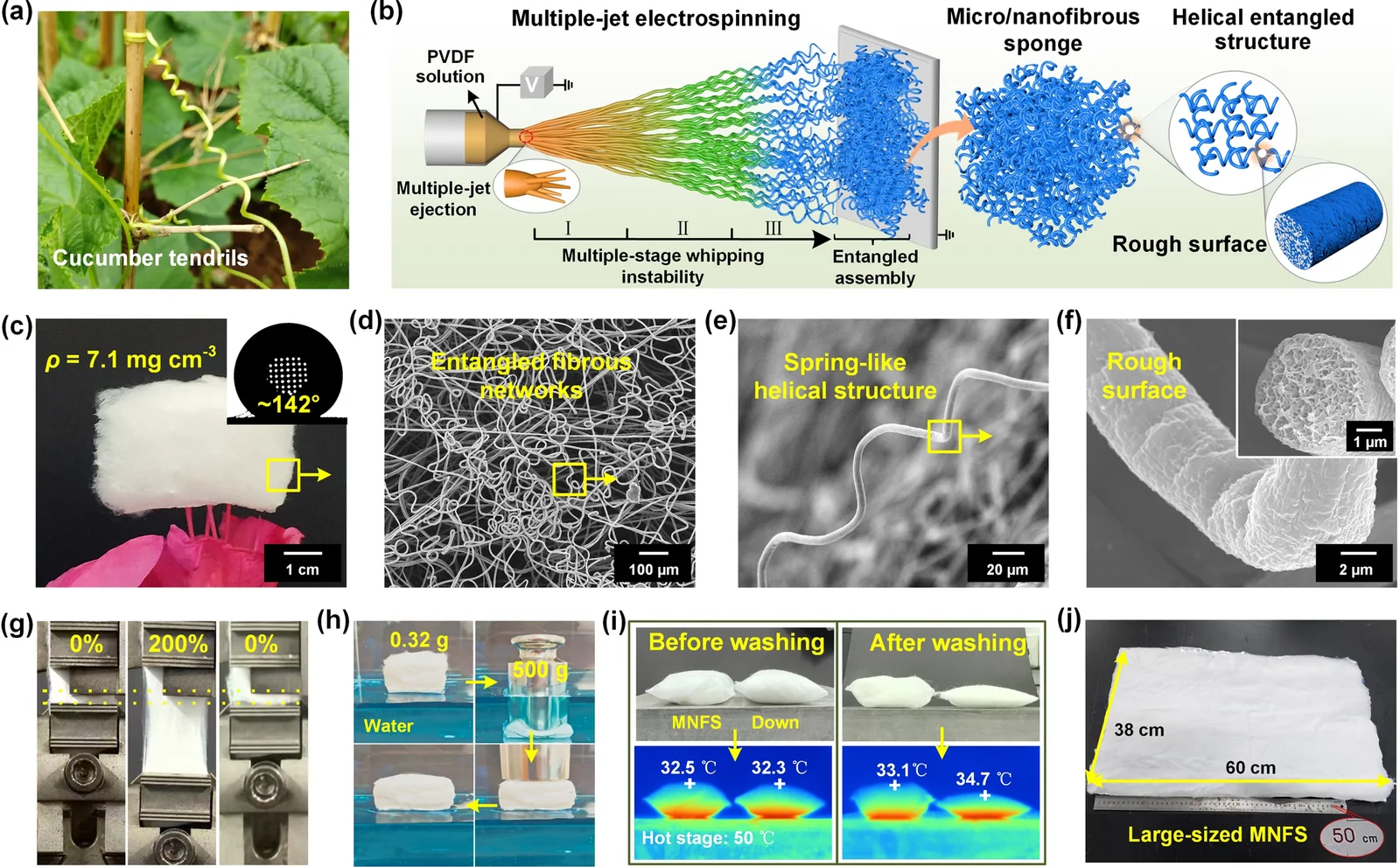
Design strategy, structure, and properties of MNFS. **a** Photographs of cucumber tendrils. **b** Schematic illustration of directly synthesis process of MNFS. **c** Optical image of MNFS self-standing on the flower stamens. Inset: photograph of a water droplet on the MNFS. **d–f** Microscopic structures for MNFS at various magnifications. **g** Demonstration of high stretchability of MNFS. **h** Compressibility of MNFS underwater. **i** Thermal insulation properties of MNFS before and after washing process. **j** Large-sized of MNFS

We developed a superelastic and washable MNFS composed of electrospun spring-like helical fibers, capable of withstanding significant mechanical stress while maintaining structural integrity and thermal insulation performance. The design of MNFS was based on two key criteria: (i) the sponge must have high porosity and small pore size to capture more stationary air and reduce heat loss and (ii) the sponge must also exhibit mechanical robustness and stable pore structure to guarantee long-term and efficient heat retention in practical applications. To fulfill these two criterions, inspired by the natural spring-like structures of cucumber tendrils (Fig. 1a), we constructed fibrous sponge consisted of biomimetic helical fibers directly during electrospinning. The spatial support of 3D helical structures of fiber results in a larger distance between the fibers, thus forming a fluffy accumulation shape at the macro level, giving the fibrous sponge a high porosity. And the second criterion was achieved by the deformation and entanglement structure of spring-like helical fibers. When subjected to external forces, the fibrous sponge dissipated the forces through the deformation of the fibers from helical to straight and the entanglement between fibers, thereby giving the fibrous sponge robust mechanical properties.

The MNFS was fabricated using multiple-jet electrospinning technology, which involved the ejection of multiple jets, the occurrence of multiple-stage whipping instability, and the formation and entangled assembly of helical fibers (Fig. 1b). By regulating the conductivity of the PVDF precursor solution, the polymer jet acquired substantial charge under high voltage, inducing multiple ejection modes and multiple-stage whipping instabilities. Furthermore, by varying the concentration of the high-volatile acetone (AC) solvent in the mixed solvent, the whipping jets underwent rapid phase separation and solidified into helical fibers featuring rough surfaces and internal porous structures. Analogous to molecular chain crosslinking during gelation, these biomimetic helical fibers interlocked directly to form an entanglement network through violent jet whipping. Ultimately, electrostatic repulsion between residual charges on the fiber surfaces created extended interfiber distances, synergizing with the spatial support of the 3D helical architecture to yield a low-density fibrous sponge. In contrast to existing fabrication strategies for fibrous sponges [24–26], this work pioneers the direct assembly of sponges composed of biomimetic spring-like helical fibers, offering a scalable and robust pathway to engineer superelastic 3D fibrous materials. The hierarchical structure with high porosity of 99.6% and pore size of 7.76 μm (Fig. S1) imparted lightweight properties to MNFS. As illustrated in Fig. 1c, a sponge with density of 7.1 mg cm^−3^ could stand gently without bending the fresh stamens. Taking advantage of the rough fiber surface, the sponge also exhibited good hydrophobicity with water contact angle (WCA) of 142° (inset of Fig. 1c). Moreover, Fig. 1d–f depicted the hierarchical microstructures of MNFS obtained by scanning electron microscope (SEM) images. The biomimetic spring-like helical fibers were interwoven to form entangled fibrous networks, providing mechanical support for the fibrous sponge. Furthermore, the tiny grooves and protrusions on the surface of fibers can provide more contact area between water droplets and fiber surface, thereby improving the non-wettability and underwater mechanical stability of fibrous sponges [27]. In addition, the internal porous structures of single fiber could suppress the free-slip of air molecules, leading to the improved thermal insulation performance [28].

In contrast to reported electrospun fibers [29, 30], the biomimetic spring-like helical fibers endow the MNFS with superelasticity, while the stable interlocking between helical fibers confers enhanced mechanical robustness. As demonstrated in Fig. 1g, MNFS exhibits remarkable stretchability, achieving a tensile strain of 200% without fracture. Moreover, MNFS also exhibited remarkable compressibility and water resistance that a 0.16 g sponge was capable of recovering its original shape after releasing the compressive stress of 3000 times its weight underwater (Fig. 1h). Beyond its mechanical durability and hydrophobicity, MNFS also demonstrated good thermal insulation capabilities due to the internal porous structures of the single fiber, small pore size, and high porosity of fibrous assemblies. Equal sized MNFS and down samples (4 cm × 4 cm × 1 cm) were encapsulated in nonwoven fabrics and then analyzed for thermal insulation using a 50 °C hot stage as a heat source. As shown in the left part of Fig. 1i, the surface temperature of MNFS was close to that of down feathers, demonstrating its superior thermal insulation capacity before washing. After washing, MNFS maintained a stable structure and a lower surface temperature (33.1 °C), whereas down feathers structurally collapsed with an increased surface temperature (34.7 °C), confirming the durability of thermal insulation capacity of MNFS (right part of Fig. 1i). Furthermore, the simplicity of electrospinning technology made it easy to fabricate MNFS on large scale. Figure 1j exhibited an optical image for MNFS with the size of 60 cm × 38 cm × 2 cm, demonstrating the scalability of the electrospinning technology. Moreover, we have fabricated MNFS with large width (~ 110 cm) by pilot electrospinning equipment, which show great potential for thermal insulation applications (Fig. S2).

### Formation and Architectures of Helical Fibers

**Fig. 2:**
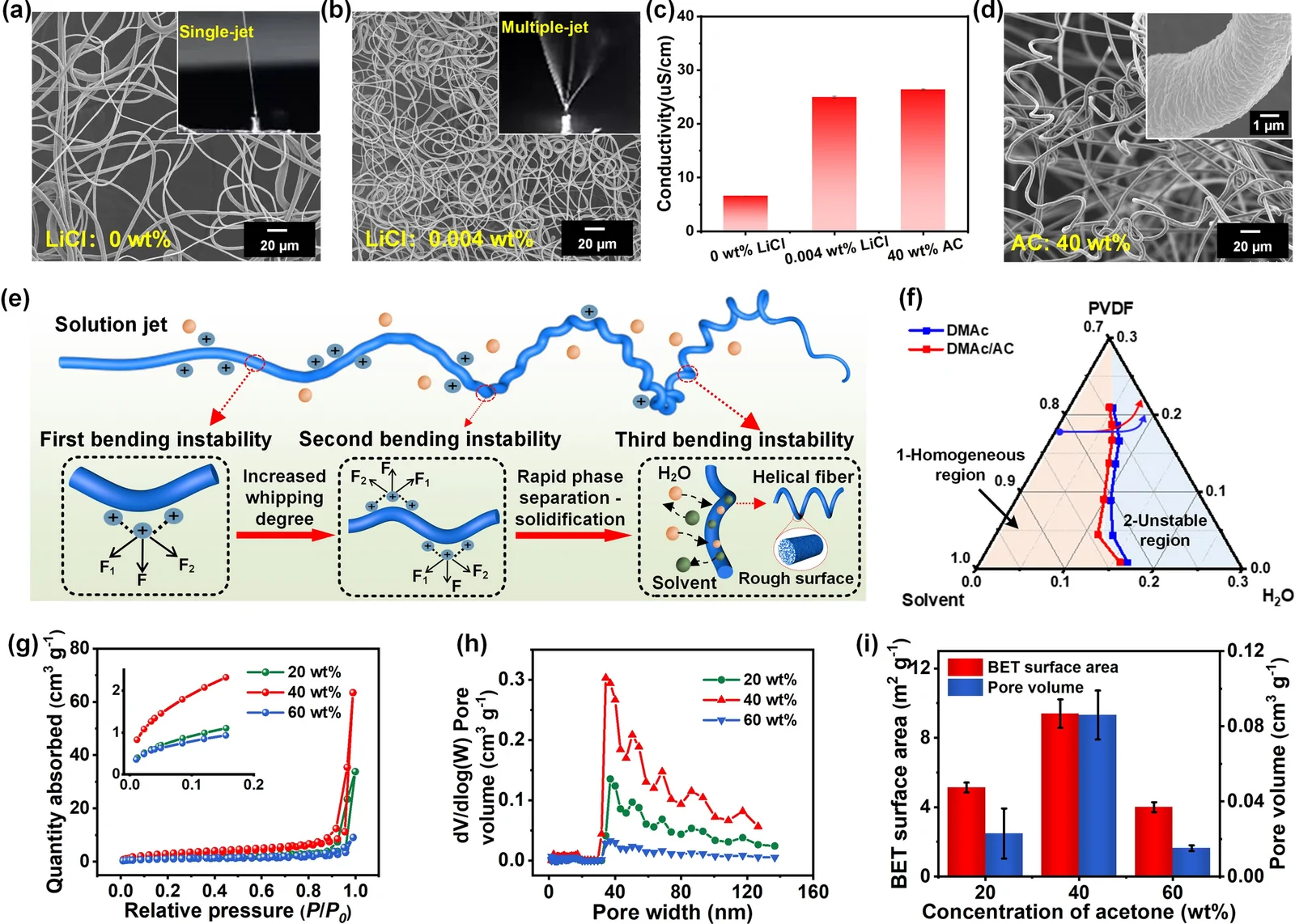
Forming regulation of helical fibers. SEM images of fibers prepared by solution containing LiCl content at **a** 0 wt% and **b** 0.004 wt%. The insets present optical photos of jet. **c** Conductivity of PVDF solution with different concentrations of LiCl (0 wt% and 0.004 wt%) and 40 wt% AC (the LiCl content was maintained at 0.004 wt%). **d** SEM images of fibers prepared by solution containing AC concentration of 40 wt%. The insets show the rough surface of fiber. **e** Formation mechanism of spring-like helical structure of fiber. **f** Ternary phase diagram for the PVDF/DMAc/H_2_O and the PVDF/DMAc/AC/H_2_O system, respectively. **g** Nitrogen adsorption/desorption curves of fibers fabricated with different AC concentrations. The inset showed the enlarge adsorption curve. **h** DFT pore size distribution, and **i** comparison for BET surface area and pore volume among fibers at different AC concentrations

The synthesis of helical fibers relied on the whipping instability and the rapid phase separation of the jets, which were closely related to the conductivity and volatilization rate of the solution, respectively. To tailor the morphology of fibers, the effect of solution conductivity on ejection mode and whipping instability of the jets was firstly studied. As shown in Fig. 2a, the solution jet with LiCl concentration of 0 wt% resulted in the single-jet ejection, further producing the straight fibers with average diameter of 2.5 μm (Fig. S3a). As the concentration of LiCl increased to 0.004 wt%, the ejection mode gradually became multiple-jet morphology and further produced the curly fibers with average diameter of 1.9 μm (Fig. S3b), which directly interlock with each other to form entangled network (Figs. S4a and 2b). Moreover, when the LiCl concentration was further increased to 0.006 wt%, the multiple-jet showed larger perturbation instability and formed straight fiber bundles (Fig. S4b). This change in fiber morphology may be due to the increased LiCl concentration increases the conductivity of the solution (Figs. 2c and S5, Table S1). When the LiCl concentration increased from 0 wt% to 0.004 wt%, the corresponding conductivity of solution increased from 6.5 to 25.0 uS cm^−1^, leading to the generation of multiple-jet and multiple-stage whipping. Subsequently, we further investigated the influence of solution volatilization rate on the morphology of bioinspired helical fibers through control of the acetone (AC) concentration in mixed solvent systems. As presented in Figs. 2c and S5, Table S1, at a fixed LiCl concentration of 0.004 wt%, increasing the AC content from 20 wt% to 60 wt% did not cause significant changes in conductivity. Surprisingly, when the AC concentration was 40 wt%, the fibers showed remarkable spring-like helical structure with rough surface (Fig. 2d). Upon increasing AC concentration to 60 wt%, the fiber morphology became straight and formed weaken rough surface (Fig. S6a). This may be caused by the increase in AC concentration, which makes the solution less viscous (Fig. S6b).

Based on the results above, a probable formation mechanism of biomimetic helical structures was demonstrated in Fig. 2e. As the jet traveled, a series of whipping instabilities and solidification phenomena occurred due to electric field-induced stretching [31]. The whipping instabilities, resulting from the competition between axial compressive and transverse bending forces, evolved through three distinct stages. First, the LiCl incorporation enhanced the ionic conductivity of solution, thereby increasing the jet’s surface charge density under high voltage. The resulting Coulomb forces (F_1_ and F_2_) redirected the jet, initiating the first bending instability [32]. Driven by the resultant force F, the jet underwent continuous bending and elongation while maintaining flight path continuity. Subsequently, solvent evaporation progressively reduced the surface charge and modified the jet’s viscoelastic properties, limiting further stretching [33]. Consequently, a secondary bending instability emerged with reduced jet diameter. In the third stage, rapid evaporation of the volatile AC solvent accelerated phase separation and jet solidification. Concurrently, the elongational viscosity of jet increased during solvent evaporation, preventing further stretching and straightening, ultimately leading to helical fiber formation [34]. Meanwhile, the rapid phase separation of jet also leads to the generation of polymer-rich and non-solvent-rich (H₂O) phases. The evaporation of the non-solvent phase forms a rough structure on fiber surface. This phenomenon could also be demonstrated by the phase diagrams of PVDF/DMAc/H_2_O and PVDF/DMAc/AC/H_2_O systems, which were constructed using the turbidity point titration method [35, 36]. The experimental cloud points exhibited a linear correlation (R^2^ > 0.98) and were positioned along the binodal curve (Fig. S7). As illustrated in Fig. 2f, the resulting ternary phase diagram comprises two distinct regions: 1-homogeneous and 2-unstable regions. When the jet crosses the binodal curve, the jet undergoes phase separation and enters the unstable region. Notably, solvent volatility governs the slope of the composition trajectory: a higher proportion of volatile AC solvent increases the trajectory slope, thereby accelerating entry into the unstable zone and promoting the formation of helical fibers with rough surfaces.

The pore structure is a critical factor determining the thermal insulation performance of fibrous sponges [37–39]. To comprehensively characterize the pore structure of the obtained fibrous assemblies, we performed nitrogen physisorption analysis. Figure 2g demonstrates that the adsorption saturation capacity of PVDF fibers increased with pore volume at AC concentrations of 20 and 40 wt%, confirming the hierarchical porous structure dominated by mesopores. Using density functional theory (DFT) calculations, we systematically analyzed the pore architecture with non-local density functional theory (NLDFT) for N_2_ adsorption at 77 K. The desorption branch was deconvoluted over a pressure range of *P*/*P*₀ = 0.01–0.99, and pore size distributions were derived by minimizing the residuals between experimental data and kernel-generated isotherms through non-negative least squares (NNLS) fitting. This analysis revealed a trimodal pore size distribution spanning three characteristic regimes: micropores (< 2 nm), mesopores (2–50 nm), and macropores (> 50 nm). Notably, fibers fabricated with 40 wt% AC exhibited higher pore density compared to other concentrations. The mesoporous domains showed a predominant size distribution peaking at 40 nm, while macroporous structures displayed a broader size dispersion ranging from 50 to 120 nm (Fig. 2h). Quantitative analysis indicated significant increases in both Brunauer–Emmett–Teller (BET) surface area (9.39 m^2^ g^−1^) and pore volume (0.09 cm^3^ g^−1^) for fibers prepared at 40 wt% AC concentrations (Fig. 2i). However, further increasing AC concentration to 60 wt% caused a marked decline in these parameters. Complementary SEM characterization (Fig. S8) corroborated the formation of hierarchical porous architectures, confirming that AC concentration modulation in mixed solvents enables controlled generation of helical fibers with rough surfaces and tunable porosity. In addition to the internal nanopores in fiber, the fibrous assemblies exhibited light weight and high porosity due to their microscale interlayer pores (Fig. S9). Additionally, the mechanical and thermal insulation properties of fiber assemblies prepared under different parameters were conducted (Fig. S10). We numbered the samples with addition of 0 wt%, 0.002 wt%, 0.004 wt%, 0.006 wt% LiCl and 40 wt%, 60 wt% AC (0.004 wt% LiCl) as 1#, 2#, 3#, 4#, 5#, 6#, respectively. The IR images in Fig. S10b demonstrated the thermal insulation performance of the fibrous assemblies. The corresponding absolute temperature different (|∆T |) was also calculated and summarized in Fig. S10c. Samples with higher |∆T | indicate better thermal insulation property. Compared with other samples, sample 5# has a greater elongation and better thermal insulation performance attributed to the entanglement helical fiber networks and 3D porous structures. Therefore, the optimal conditions for preparing the helical fibers are 0.004 wt% LiCl and 40 wt% AC.

### Mechanical Properties of MNFS

**Fig. 3:**
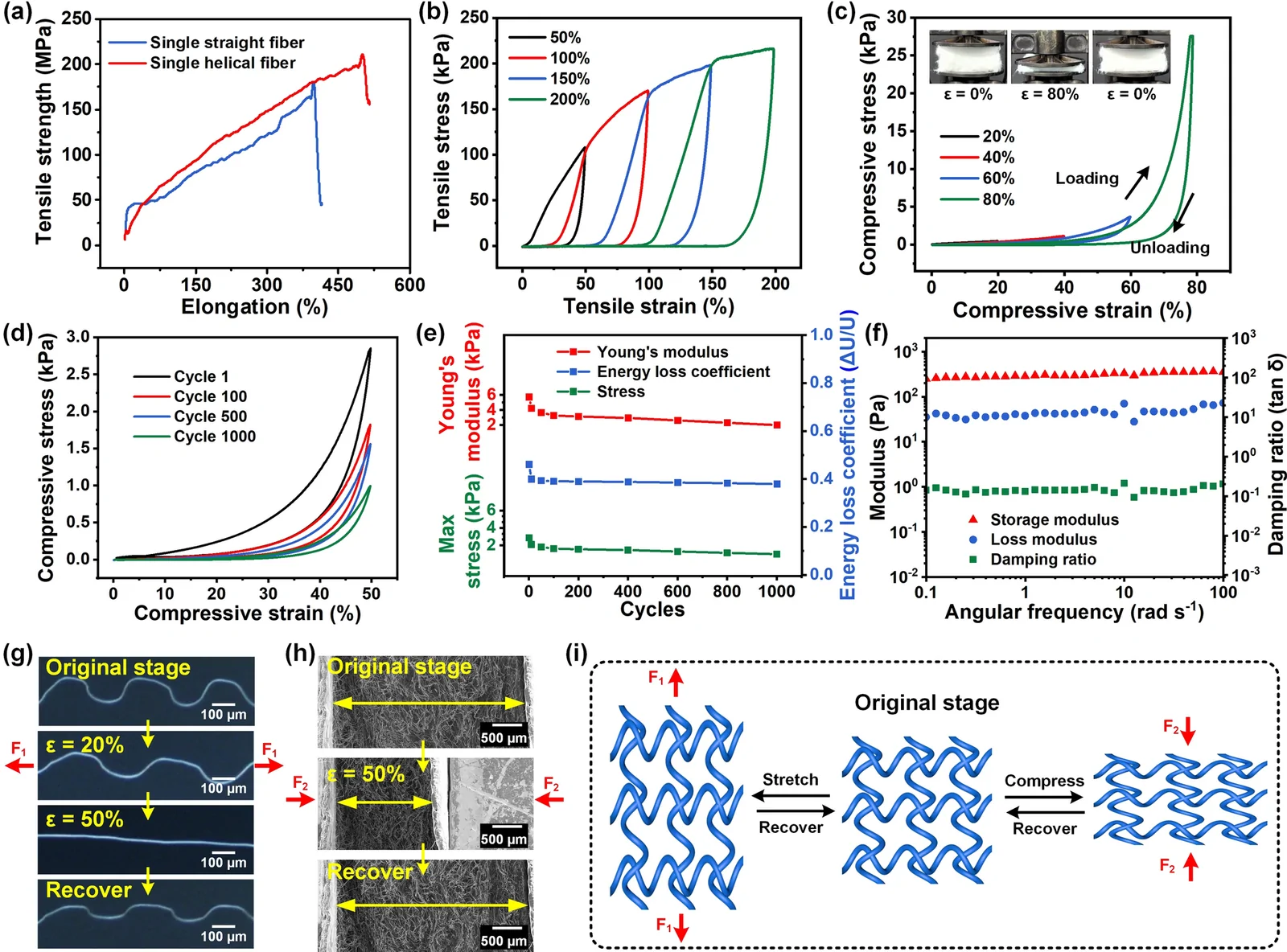
Mechanical characteristics of MNFS. **a** Elongation and tensile strength of single straight fiber and helical fiber. **b** Tensile stress–strain curves of MNFS as strain increased from 50% to 200%. **c** Compression stress–strain curves for MNFS as strain amplitude increased. Insets show transformation for MNFS at 80% compression strain. **d** A 1000 cycle compressive fatigue test at 50% strain. **e** Maximum stress, Young’s modulus, and energy loss coefficient for MNFS under various cycles. **f** Storage modulus, loss modulus, and damping ratio for MNFS at frequencies ranging from 0.1 to 100 rad s^−1^. **g** Stretch-recovery behavior of helical fiber and **h** Compressive-recovery behavior of MNFS observed via in situ SEM. **i** Corresponding schematic diagram of MNFS during tensile and compression process

We compared the tensile fracture strength between single straight fibers and helical fibers to evaluate the mechanical properties of MNFS (Movie S1). The helical fiber exhibited a large elongation of approximately 500%, which was 1.2 times greater than straight fiber (~ 400%), with corresponding tensile strength of 210 and 175 MPa, respectively (Fig. 3a). As shown in Fig. 3b, MNFS could withstand a large tensile strain of 200% without fracture at a maximum stress of 216 kPa, which benefits from the multilevel reinforcement mechanism of entangled helical fiber networks. Moreover, MNFS retained 58% of maximum stress after 1000 stretching cycles at 50% strain (Fig. S11a and Movie S2). The MNFS also exhibited good compression properties, withstanding 80% compressive strain while maintaining complete elastic recovery (Fig. 3c). Cyclic compression tests at 50% strain revealed minimal plastic deformation after 1000 cycles (Fig. 3d), with no significant degradation in maximum stress, Young’s modulus, or energy loss coefficient (Fig. 3e), confirming excellent fatigue resistance. Dynamic mechanical analysis showed stable loss modulus, storage modulus, and damping ratio across frequency sweep of 0.1 to 100 rad s^−1^ (Fig. 3f), highlighting robust viscoelastic performance. Remarkably, the MNFS maintained instantaneous shape recovery even when compressed in liquid nitrogen (Fig. S11b), demonstrating good mechanical resilience in cryogenic environments. Compared with other thermal insulation materials (Table S2), MNFS presented good mechanical properties, including tensile strength of 216 kPa at 200% strain and compressive strength of 27 kPa at 80% strain.

To elucidate the elastic mechanism of MNFS, we carried out in situ tensile and compressive tests on individual helical fibers and fibrous sponges. The helical fibers exhibited remarkable reversible deformation, achieving 50% tensile strain with a proportional increase in helical pitch. Instantaneous pitch recovery upon strain release confirmed the superelasticity of PVDF helical fibers, attributed to their spring-like microstructure (Fig. 3g). Furthermore, the MNFS demonstrated exceptional shape recovery after 50% compressive strain (Fig. 3h), revealing the structural stability of its entangled helical network. These outstanding mechanical properties are all attributed to the hierarchical structural reinforcement in MNFS. Figures 3i and S12 schematically illustrate the strengthening mechanism of MNFS. Under external loading, stress dissipation proceeds through three synergistic steps: (i) Primary deformation: The 3D porous structure deforms to dissipate initial stress; (ii) Stress redistribution: Entanglement points within the fiber network redistribute multi-axial forces, enabling cooperative load-bearing among the fibers; and (iii) Energy dissipation: Helical fiber elongation and conformational changes resist fracture. The integration of these structures endows MNFS with exceptional mechanical robustness.

### Thermal Insulation Performance for MNFS

**Fig. 4:**
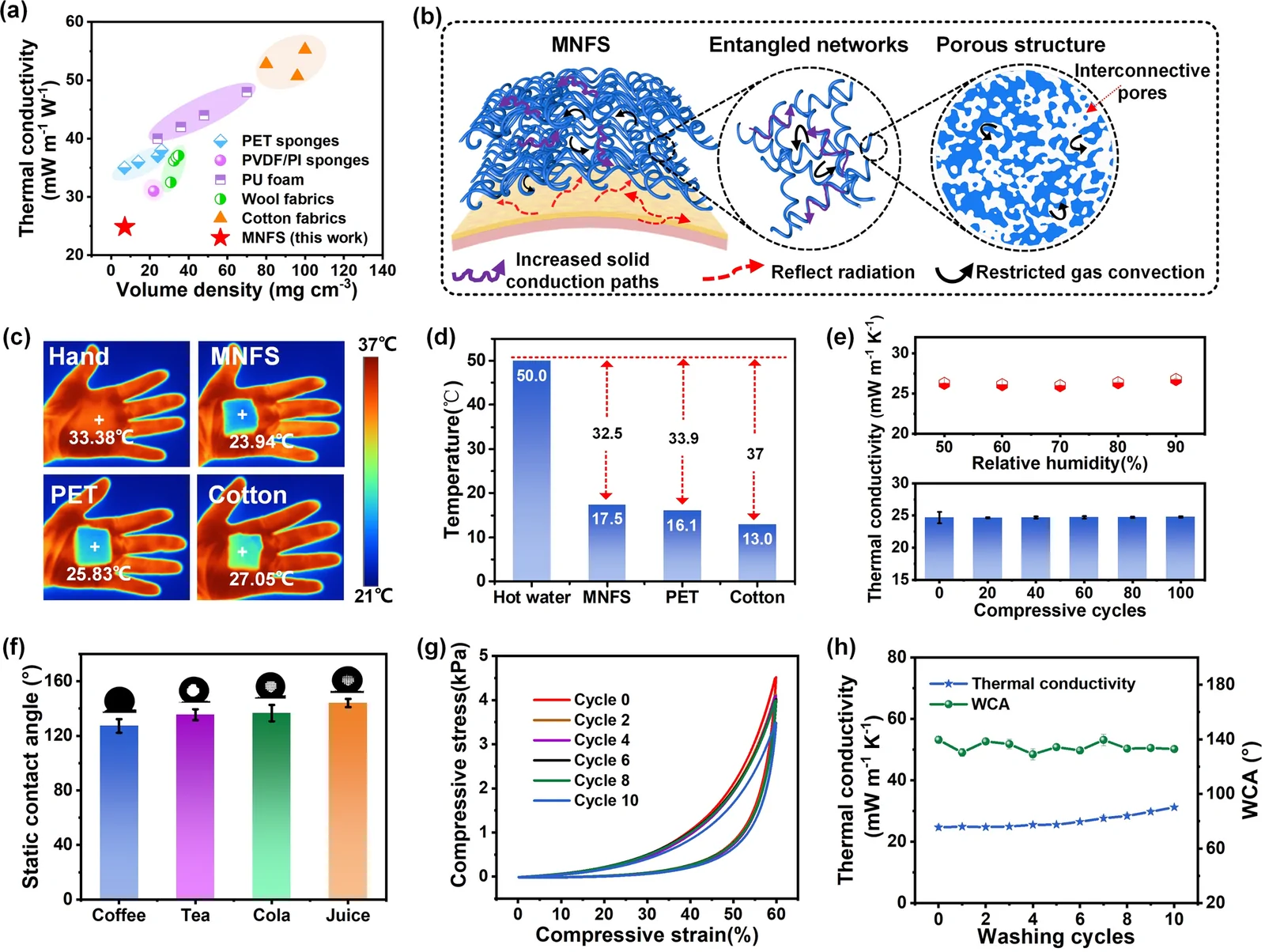
Thermal insulation properties and washability of MNFS. **a** Thermal conductivity and density for fluffy fibrous materials reported in literature. **b** Schematic of thermal insulation mechanism of MNFS. **c** Infrared images of human palm under various coverings. **d** Temperature of hot water in a tube covered with different thermal insulation materials in cold air. **e** Thermal conductivity of MNFS under the environment with diverse relative humidity (top) and various compression cycles (bottom), respectively. **f** Different liquid contact angles of MNFS. **g** Compressive stress–strain of MNFS under various washing cycles. **h** Thermal conductivities and WCAs of MNFS under different washing cycles

We further investigated the thermal insulation capacity of MNFS. Generally, the total heat transfer (*λ*_total_) of porous materials depends on the thermal property of material composition and structure of aggregates (such as pore size and porosity) [40]. The *λ*_total_ can be expressed as a sum of solid thermal conduction, gas thermal conduction, thermal convection, and thermal radiation [41, 42]. The obtained MNFS exhibits high porosity, small pore size, and lightweight characteristics, enabling high-efficiency thermal insulation in low-temperature environments. Compared to typical thermal insulation materials [43–47], the MNFS demonstrated superior performance in terms of both light weight and low thermal conductivity (Fig. 4a and Table S2). As can be seen in Table S2, our PVDF micro/nanofibrous sponge exhibited remarkable thermal insulation performance, achieving a low thermal conductivity (*λ*) of 24.85 mW m^−1^ K^−1^, comparable to the value of dry air [48, 49], along with a low density of 7.1 mg cm^−3^. These properties surpass those of most reported fibrous thermal insulating materials. To provide insight into the heat transfer mechanisms of conventional electrospun straight fiber assembly and MNFS, we conducted 3D simulations. As shown in Fig. S13, the comparative temperature distribution analysis demonstrates that MNFS exhibits superior thermal insulation capability compared to conventional electrospun fiber assembly. Figure 4b schematically illustrates the suppressed heat transfer mechanisms in MNFS: (i) The entangled helical fiber networks prolong the heat transfer pathways, reducing solid-phase thermal conduction; (ii) The small pores within individual fibers restrict gas molecule motion and collisions (*Knudsen effect*) [50], significantly suppressing gas-phase convection and conduction. Further evidence of MNFS’s thermal insulation performance is shown in Fig. 4c, which compares infrared images of a human palm and palms covered with MNFS, PET felt, or cotton felt (all 10 mm thick). Notably, the MNFS sample exhibited lower surface temperatures in infrared imaging, with a temperature difference (ΔT) of 9.44 °C relative to the bare palm, which was higher than those of PET (7.55 °C) and cotton (6.33 °C), respectively. The higher ΔT means the better thermal insulation capability. In additional, we simulated the thermal insulation property of materials in cold air. Using a 50 °C heated water-filled tube as a simulated heat source (device schematic in Fig. S14), uniformly sized (5 cm × 5 cm × 1 cm) material samples were used to encapsulate the tube surface and then exposed to 3 °C ambient air. Temperature monitoring via thermocouples revealed that slower cooling rates directly reflected good thermal insulation capacity after 30 min [51]. As shown in Fig. 4d, the ΔT of MNFS was 32.5 °C, lower than those of PET (33.9 °C) and cotton (37 °C), further confirming the better thermal insulation capacity of MNFS. We also assessed the thermal insulation capacity of MNFS under various environmental conditions. The infrared images in Fig. S15 demonstrated the thermal insulation performance of MNFS at stage temperatures of − 22, 0, and 50 °C. MNFS could maintained a high surface temperature of 23.6 °C at − 22 °C, confirming its efficacy in extreme cold environments. Figure 4e shows the *λ* of MNFS after different compressive cycles and at various relative humidity. The *λ* of MNFS remained constant throughout the entire 100 compression cycles. Moreover, the *λ* remained stable even when the ambient relative humidity was increased.

In addition to its stable thermal insulation performance in high-humidity environments, MNFS exhibited desirable liquid-repellent properties, as reflected by the contact angles of coffee (127°), tea (135°), cola (136°), and juice (144°) (Fig. 4f). Meanwhile, these liquids could also be easily removed from the MNFS surface (Fig. S16). Benefiting from the good hydrophobicity and robust mechanical properties, MNFS also exhibits remarkable washability. As shown in Fig. 4g, after 10 washing cycles, the compressive stress and strain remained at 3.48 kPa and 60%, respectively, retaining 77% of the maximum stress observed before washing. And its tensile stress of the MNFS could also maintain over 84% of the original value (Fig. S17). These results indicated that the entangled helical fiber networks were stable in the washing process. Moreover, MNFS maintained a static WCA of approximately 140° throughout the washing cycles, while its thermal conductivity remained nearly constant (Fig. 4h). Even after 60 washing cycles, the thermal conductivity experienced a slight increase to 33.3 mW m^−1^ K^−1^ (Fig. S18). This minor change demonstrates the retention of structural integrity during the repeated washing process. Additionally, UV-resistance testing and outdoor exposure experiments were conducted at temperatures of 26–35 °C for 1–4 days. As shown in Fig. S19 and Table S3, the UPF of MNFS exceeds 50 + (UVA transmittance < 0.1%), confirming excellent UV resistance. The superior UV resistance of MNFS arises from (i) multiple reflections of UV radiation within its entangled fiber network and porous architecture and (ii) effective blocking of UV penetration through the material thickness. The outdoor experiments revealed no yellowing or aging of MNFS, while mechanical properties and thermal insulation remained stable, demonstrating the reliability and durability of MNFS (Fig. S20). The synergistic combination of versatility positions MNFS as a robust candidate for application in aerospace, transportation, and building insulation that demand both thermal efficiency and practical maintenance (Table S4).

## Conclusions

In summary, we successfully fabricated superelastic and washable sponges based on biomimetic spring-like helical micro/nanofibers for efficient thermal insulation using multiple-jet electrospinning technology. The ejection mode, multiple-stage whipping instability, and rapid phase separation of the charged jets were tailored by controlling the concentration of LiCl and AC solvent, leading to the formation of spring-like helical fibers that directly entangled with each other to assemble micro/nanofibrous sponges. The resultant MNFS exhibited a hierarchical porous structure with porosity of 99.6% and lightweight with density of 7.1 mg cm^−3^. The synergistic effect of single helical fibers and their entanglement network endowed MNFS with remarkable mechanical robustness, including a large tensile strain of 200%, resistance to 1000 cyclic tensile or compression deformations, and stability under low-temperature conditions. Additionally, the MNFS demonstrated efficient heat retention capabilities with low thermal conductivity of 24.85 mW m^−1^ K^−1^, comparable to dry air, and maintained structural stability even after cyclic washing. This study provides valuable insights into the design of advanced fibrous sponges and demonstrates their potential for applications in aerospace, building insulation, and transportation.

## Supplementary Information

Below is the link to the electronic supplementary material.

Supplementary file1 (MP4 1748 KB)

Supplementary file2 (MP4 2148 KB)

Supplementary file3 (DOCX 1544 KB)
