# Supplementary material for: Superelastic and Washable Micro/Nanofibrous Sponges Based on Biomimetic Helical Fibers for Efficient Thermal Insulation
Source: Nanomicro Lett. 2025 Aug 25;18:42. doi: 10.1007/s40820-025-01882-2 (PMC12379200; doi:10.1007/s40820-025-01882-2)
Supplement: Supplementary file 3 — Supplementary file3 (DOCX 1544 KB) [file 40820_2025_1882_MOESM3_ESM.docx]

Supporting Information for

**Superelastic and Washable Micro/Nanofibrous Sponges Based on Biomimetic Helical Fibers for Efficient Thermal Insulation**

Fengjin Yang^1^, Zhifei Wang^1^, Wei Zhang^1^, Sai Wang^1^, Yi-Tao Liu^1^, Fei Wang^1,^ *, Roman A. Surmenev^3^, Jianyong Yu^1^, Shichao Zhang^1^, and Bin Ding^1, 2^

^1^ Innovation Center for Textile Science and Technology, College of Textiles, Donghua University, Shanghai 200051, P. R. China

^2^ School of Materials Science and Engineering, Shanghai University of Engineering Science, Shanghai 201620, P. R. China

^3^ Physical Materials Science and Composite Materials Center, Research School of Chemistry & Applied Biomedical Sciences, National Research Tomsk Polytechnic University, Tomsk 634050, Russia

*Corresponding author. E-mail: [feiwang@dhu.edu.cn](mailto:feiwang@dhu.edu.cn) (Fei Wang)

**S1 Supplementary Figures**


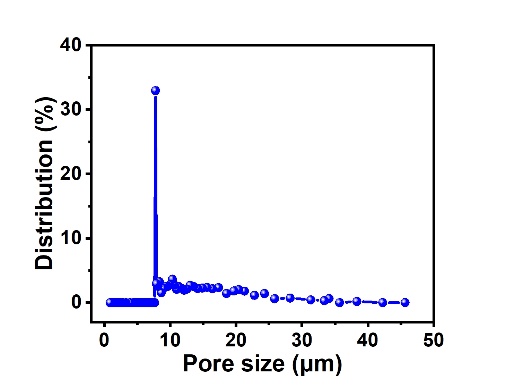


**Fig. S1** Pore size distribution of MNFS prepared at the LiCl concentration of 0.004 wt% and AC concentration of 40 wt%


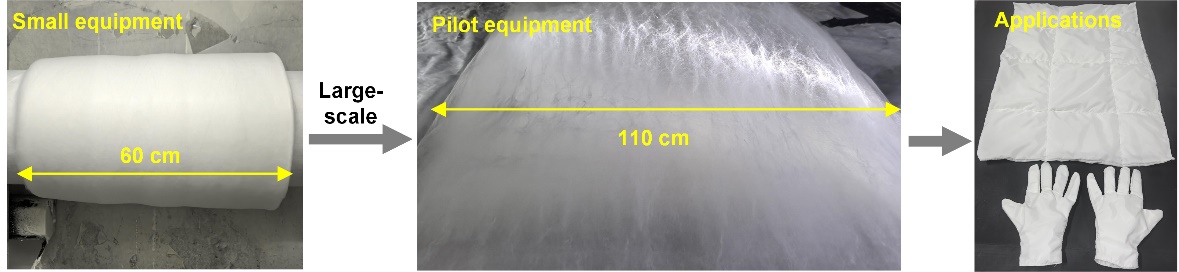


**Fig. S2** The scalability of our strategy


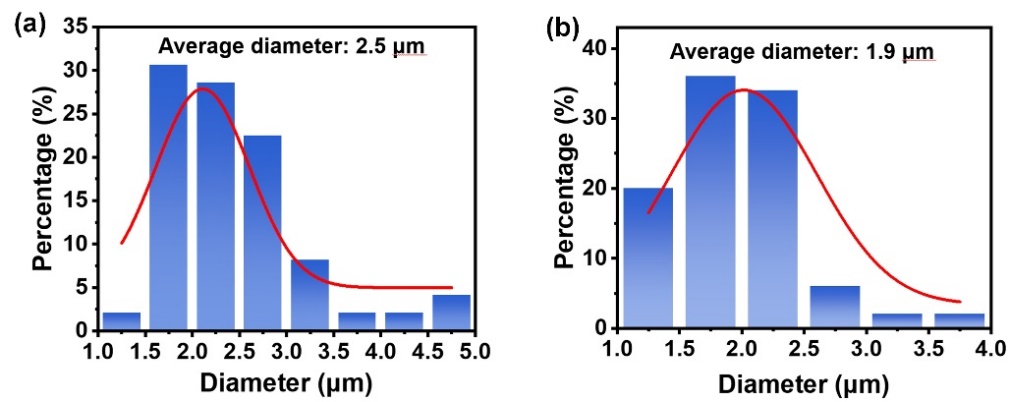


**Fig. S3** Diameter distribution diagram of PVDF fibers. Diameter of fibers prepared by solution containing LiCl content at **a** 0 wt% and **b** 0.004 wt%


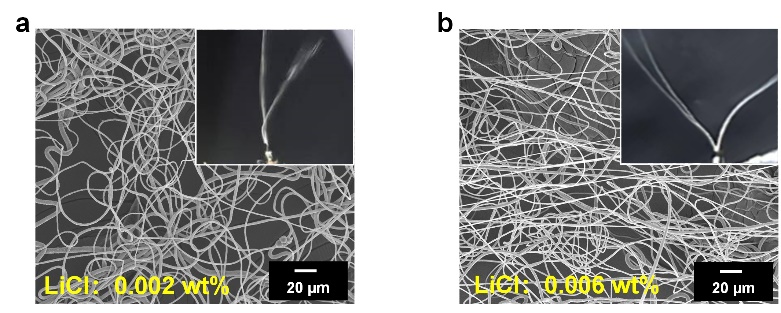


**Fig. S4** SEM images of the fibers prepared from PVDF solution with the LiCl content of **a** 0.002 wt% and **b** 0.006 wt%. The insets present the optical photos of the jets.


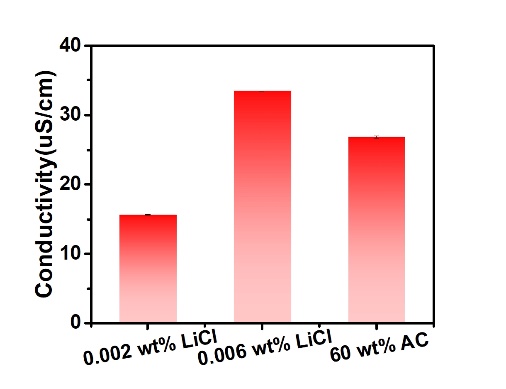


**Fig. S5** Conductivity of PVDF solution with different concentration of LiCl (0.002 and 0.006 wt%) and AC (60 wt%)


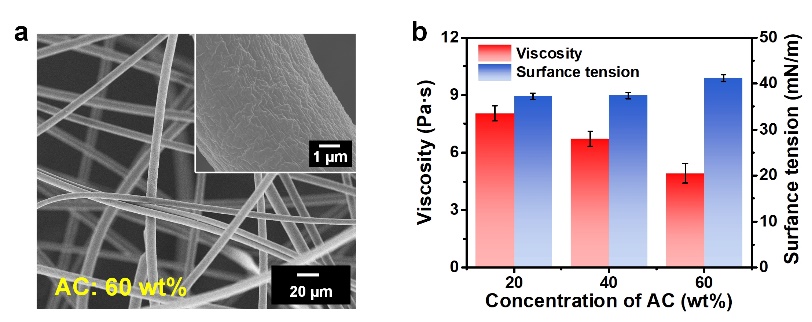


**Fig. S6** **a** SEM images of the fibers prepared from PVDF solution with acetone concentration of 60 wt%. The insets present the SEM images of surface mophology of the single fiber. **b** Surface tension and viscosity of PVDF solution with different AC concentration (20, 40, and 60 wt%).


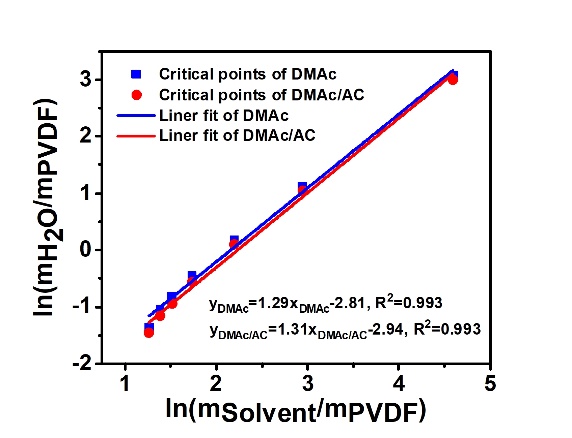


**Fig. S7** Linearized cloud point plots of the PVDF/DMAc/H_2_O and PVDF/DMAc/AC/H_2_O system


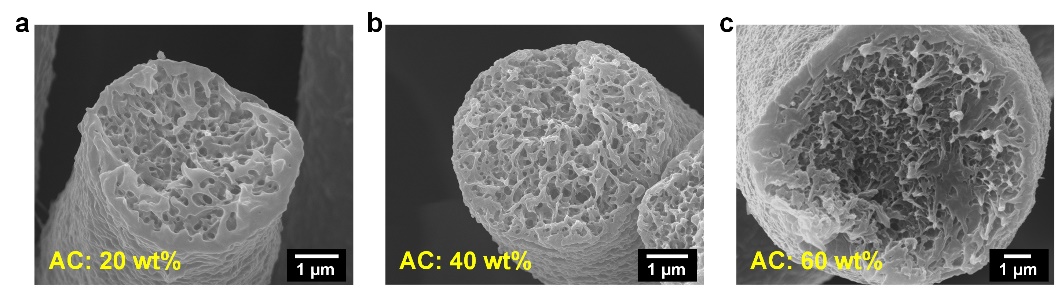


**Fig. S8** Cross-section images of porous structures under different concentration of AC. **a** 20, **b** 40, and **c** 60 wt%.


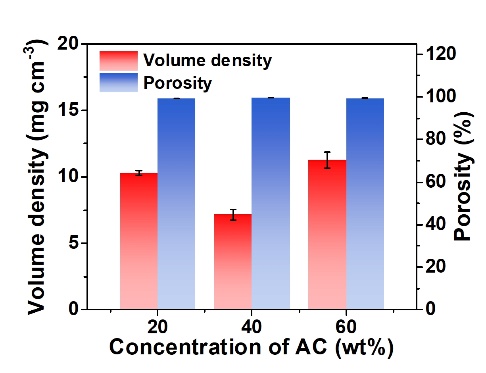


**Fig. S9** The porosity and volume density of fiber assemblies prepared from the solution with different AC concentration.


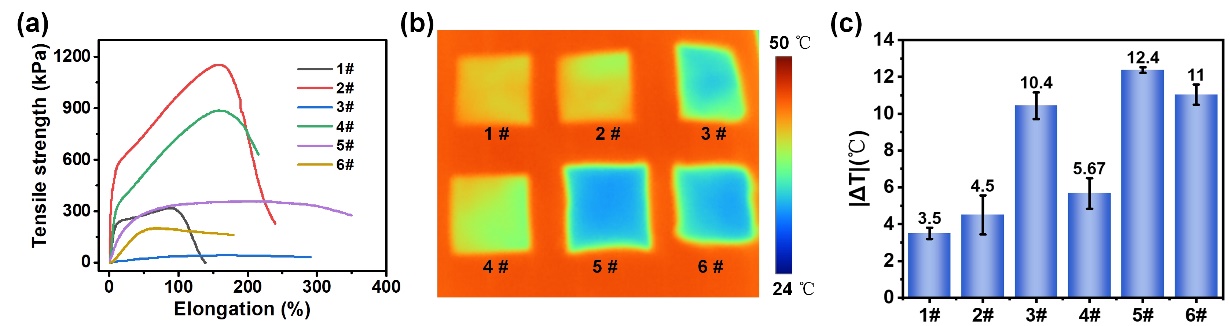


**Fig. S10** Mechanical and thermal insulation performance of fibrous assemblies prepared under different concentration of LiCl and AC. **a** Elongation and tensile strength of fibrous assemblies. **b** IR images of fibrous assemblies placed on a heating stage (50 ℃). **c** Temperature difference (|ΔT |) between the sample surface and the stage


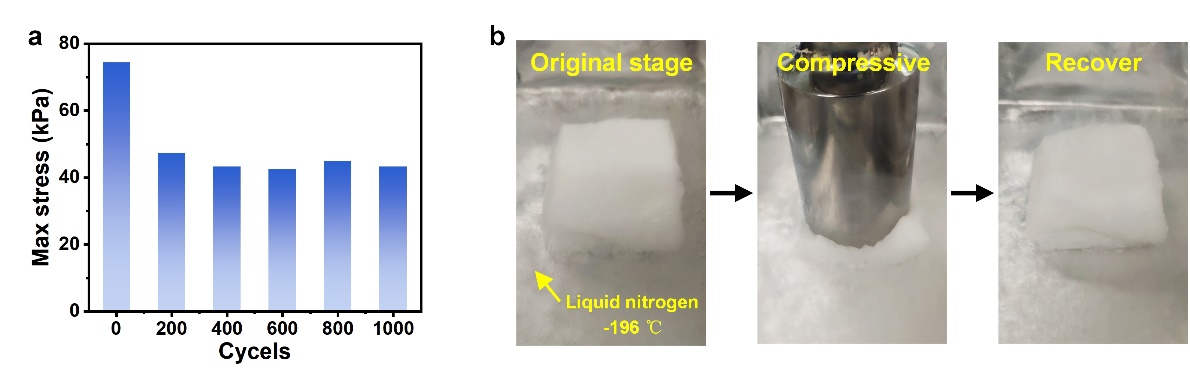


**Fig. S11** **a** Max stress of MNFS under different cycles with strain of 50%. **b** Presentation showing that MNFS could maintain resilience in liquid nitrogen


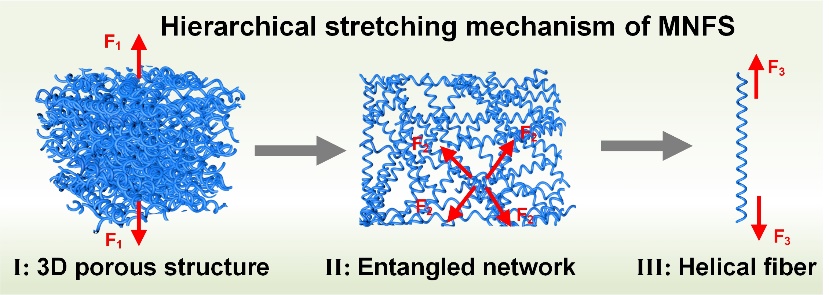


**Fig. S12** The tensile fracture mechanism of MNFS


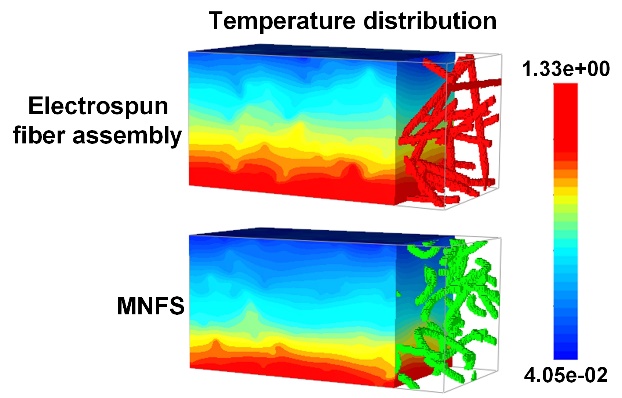


**Fig. S13** The thermal transfer behavior models of electrospun fiber assembly and MNFS


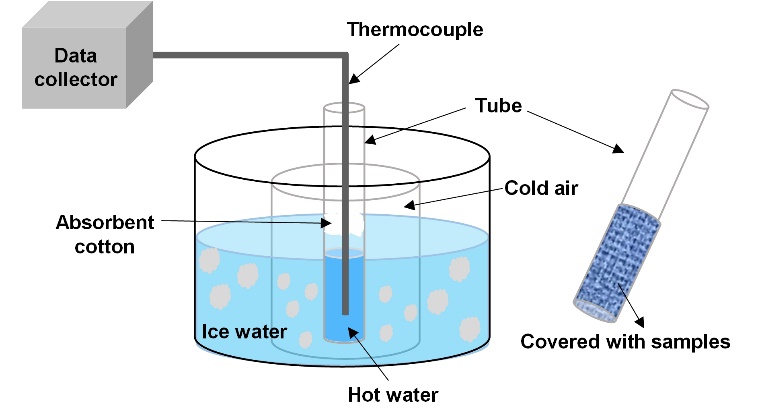


**Fig. S14** Schematic illustration of the set-up for thermal insulation test


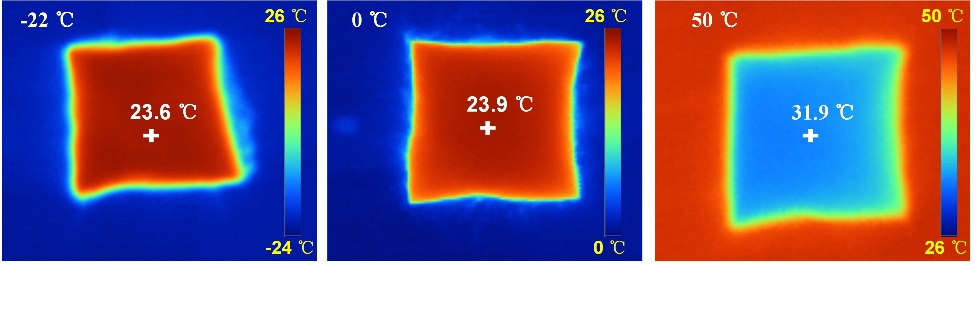


**Fig. S15** Thermal insulation performance of MNFS under different temperature conditions


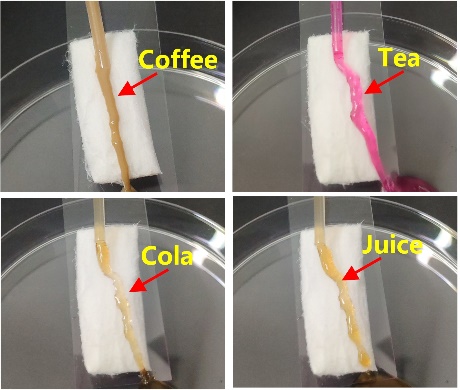


**Fig. S16** Photographs showing the liquid-repellent properties of the MNFS


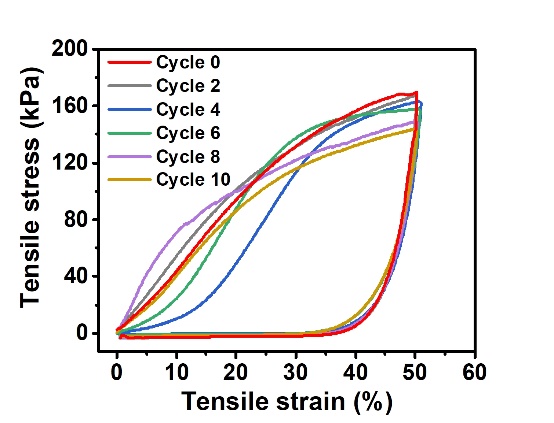


**Fig. S17** Tensile stress-strain of MNFS under various washing cycles


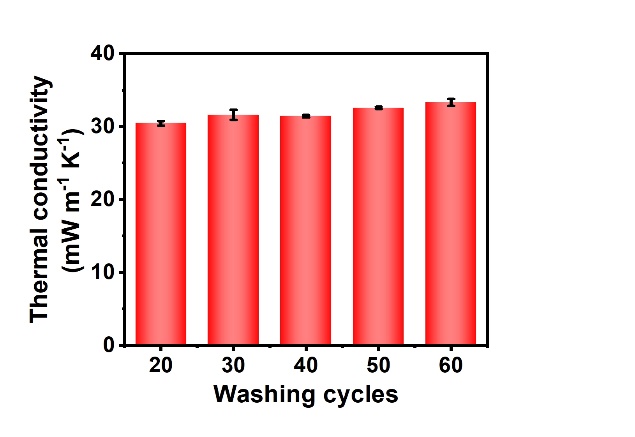


**Fig. S18** Thermal conductivity of MNFS under various washing cycles


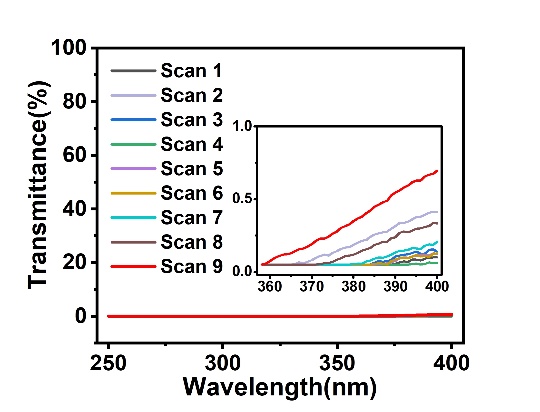


**Fig. S19** The ultraviolet transmittance of MNFS


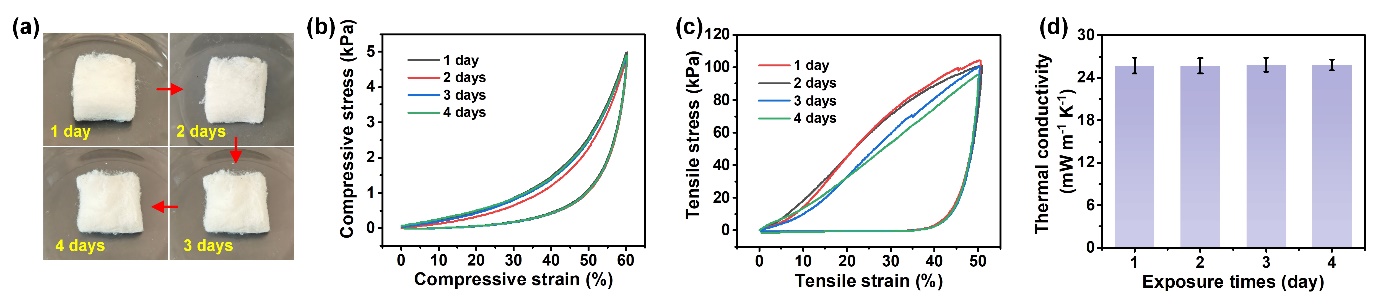


**Fig. S20** The reliability and durability of MNFS. **a** morphology, **b** Compressive stress-strain, **c** Tensile stress-strain, and d thermal conductivity of MNFS under different exposure times

**S2 Supplementary Tables**

**Table S1** The conductivity of the solution with different LiCl concentration and AC content

| LiCl  [wt%] | AC  [wt%] | DMAc  [wt%] | Conductivity  [μS/cm] |
| --- | --- | --- | --- |
| 0 | 20 | 80 | 6.5±0.02 |
| 0.002 | 20 | 80 | 15.6±0.05 |
| 0.004 | 20 | 80 | 25.0±0.17 |
| 0.006 | 20 | 80 | 33.5±0.1 |
| 0.004 | 40 | 60 | 26.3±0.16 |
| 0.004 | 60 | 40 | 26.8±0.2 |

**Table S2** The comparison of the density, thermal conductivity, and mechanical properties for the commercial and reported thermal insulating materials

| Component | Synthesis strategy | | Density  [mg cm^-3^] | | Thermal  conductivity  [mW m^-1^ K^-1^] | Tensile strength [kPa] | Compressive  strength  [kPa] | | Reference |
| --- | --- | --- | --- | --- | --- | --- | --- | --- | --- |
| Commercial Cotton | | / | | 27.6 | 46.31 | 16.6 (50% strain) | 8.74 (70% strain) | Self testing | |
| Commercial Polyester | | / | | 10.4 | 42.33 | 10.8 (50% strain) | 6.25 (80% strain) | Self testing | |
| PI | | Freeze-drying | | 26 | 30.06 | 160 (25% strain) | ~160 (90% strain) | [1] | |
| CNF/PPy/PU | |  |  | 140-160 | 52-56 | None | None | [2] | |
| Carbon/SiO_2_  @CNTs | |  |  | 40 | ~48 |  | 17.65 (75% strain) | [3] | |
| SiBCN/SiC | |  |  | 142 | 52 |  | None | [4] | |
| Sodium alginate/ Al_2_O_3_ | |  |  | 53 | 27.2 |  | 2100 (80% strain) | [5] | |
| PPS | |  |  | 6.4 | 28 | 8 (25% strain) | ~120 (95% strain) | [6] | |
| BSiTa-PA | | Ambient pressure drying | | 180 | 49.6 | None | 768.7 (30% strain) | [7] | |
| Zircon | | Airflow- assisted spinning | | 20 | 26 | 42.4 (18.5% strain) | ~90 (95% strain) | [8] | |
| Polyimide | |  |  | 50 | 37 | None | 15 (80% strain) | [9] | |
| Yttria-stabilized zirconia | |  |  | 65 | 21 |  | ~5 (50% strain) | [10] | |
| Mullite | | Electrospinning | | 8 | 20 | 3.5 (20% strain) | ~3.7 (80% strain) | [11] | |
| Zirconia-silica | | Off-axial electrospinning | | 8 | 24.6 | ~45 (150% strain) | 35 (90% strain) | [12] | |
| PVDF | | Multiple-jet electrospinning | | 7.1 | 24.85 | 216 (200% strain) | 27 (80% strain) | This work | |

**Table S3** The UV-resistance performance of MNFS

|  | UPF | T(UVA)/% | T(UVB)/% |
| --- | --- | --- | --- |
| Number of Scans | 9 | 9 | 9 |
| Mean | 1937.06 | 0.09 | 0.05% |
| STD | 89.45 | 0.05% | 0.00% |
| COV | 4.62% | 57.51% | 0.00% |

**Table S4** Performance requirement for difference application scenarios

| Application scenarios | Component | Project | Index requirements | | References |
| --- | --- | --- | --- | --- | --- |
| Aerospace | Cargo insulated containers | Working environments | Temperature (-30-45℃), relative humidity (100%) | | GB/T 18433-2023 |
| Transportation | Refrigerated trailers | Thermal conductivity (25℃)/[mW m^-1^ K^-1^] | ≤24 | | GB/T 40363-2021 |
| Building insulation | External Wall Insulation Modules | Thermal conductivity (25℃)/[mW m^-1^ K^-1^] | Grade 024 | ≤24 | GB/T 30595-2024 |
|  |  |  | Grade 030 | ≤30 |  |
|  |  |  | Grade 034 | ≤34 |  |

**S3 Supplementary Captions for Movies S1 to S2**

Movie S1: Demonstration of tensile fracture process of single helical fiber.

Movie S2: Demonstration of tensile and recovery process of single helical fiber.

**Supplementary References**

1. Y. Pan, J. Zheng, Y. Xu, X. Chen, M. Yan et al., Ultralight, highly flexible *in situ* thermally crosslinked polyimide aerogels with superior mechanical and thermal protection properties *via* nanofiber reinforcement. J. Colloid Interface Sci. **628**, 829–839 (2022). <https://doi.org/10.1016/j.jcis.2022.07.144>
2. Y. Wang, L. Chen, H. Cheng, B. Wang, X. Feng et al., Mechanically flexible, waterproof, breathable cellulose/polypyrrole/polyurethane composite aerogels as wearable heaters for personal thermal management. Chem. Eng. J. **402**, 126222 (2020). <https://doi.org/10.1016/j.cej.2020.126222>
3. X. Wang, Y. Yuan, X. Sun, R. Qiang, Y. Xu et al., Lightweight, flexible, and thermal insulating carbon/SiO_2_@CNTs composite aerogel for high-efficiency microwave absorption. Small **20**(30), 2311657 (2024). <https://doi.org/10.1002/smll.202311657>
4. J. Jiang, L. Yan, Y. Xue, J. Li, C. Zhang et al., Lightweight and thermally insulating polymer-derived SiBCN/SiCnw ceramic aerogel with enhanced electromagnetic wave absorbing performance. Chem. Eng. J. **482**, 148878 (2024). <https://doi.org/10.1016/j.cej.2024.148878>
5. H. Xu, C. Liu, W. Guo, N. Li, Y. Chen et al., Sodium alginate/Al_2_O_3_ fiber nanocomposite aerogel with thermal insulation and flame retardancy properties. Chem. Eng. J. **489**, 151223 (2024). <https://doi.org/10.1016/j.cej.2024.151223>
6. Y. Yu, C. Xu, Z. Hu, H. Xiang, J. Zhang et al., Industrial scale sea-island melt-spun continuous ultrafine fibers for highly comfortable insulated aerogel felt clothing. Adv. Mater. **36**(52), 2414731 (2024). <https://doi.org/10.1002/adma.202414731>
7. Z. Niu, F. Qu, F. Chen, X. Ma, B. Chen et al., Multifunctional integrated organic-inorganic-metal hybrid aerogel for excellent thermal insulation and electromagnetic shielding performance. Nano-Micro Lett. **16**(1), 200 (2024). <https://doi.org/10.1007/s40820-024-01409-1>
8. J. Guo, S. Fu, Y. Deng, X. Xu, S. Laima et al., Hypocrystalline ceramic aerogels for thermal insulation at extreme conditions. Nature **606**(7916), 909–916 (2022). <https://doi.org/10.1038/s41586-022-04784-0>
9. Y. Liu, X. Pan, Z. Zhou, Y. Xiao, H. Mei et al., Ultralight and elastic polyimide microtube aerogel *via* airflow-induced spinning. Adv. Mater. 2503499 (2025). <https://doi.org/10.1002/adma.202503499>
10. L. Su, J. Lu, H. Zhang, X. Li, J. Wang et al., An ultralight all-fiber-structure sponge with thermal and electromagnetic integrated insulation property. J. Am. Ceram. Soc. **107**(9), 5993–6003 (2024). <https://doi.org/10.1111/jace.19902>
11. X. Chang, X. Cheng, X. Yin, R. Che, J. Yu et al., Multimode thermal gating based on elastic ceramic-carbon nanowhisker/nanofiber aerogels by strain engineering strategy. ACS Nano **19**(10), 10421–10432 (2025). <https://doi.org/10.1021/acsnano.5c00125>
12. S. Dang, J. Guo, Y. Deng, H. Yu, H. Zhao et al., Highly-buckled nanofibrous ceramic aerogels with ultra-large stretchability and tensile-insensitive thermal insulation. Adv. Mater. **37**(4), 2415159 (2025). <https://doi.org/10.1002/adma.202415159>
